# Lipoxygenase catalyzed metabolites derived from docosahexaenoic acid are promising antitumor agents against breast cancer

**DOI:** 10.1038/s41598-020-79716-x

**Published:** 2021-01-11

**Authors:** Kun-Ming Chen, Henry Thompson, John P. Vanden-Heuvel, Yuan-Wan Sun, Neil Trushin, Cesar Aliaga, Krishne Gowda, Shantu Amin, Bruce Stanley, Andrea Manni, Karam El-Bayoumy

**Affiliations:** 1 Department of Biochemistry and Molecular Biology, College of Medicine, Pennsylvania State University, Hershey, PA 17033 USA; 2 Cancer Prevention Laboratory, Colorado State University, Fort Collins, CO 85023 USA; 3 Department of Veterinary and Biomedical Sciences, Pennsylvania State University, University Park, PA 16802 USA; 4 Department of Pharmacology, Pennsylvania State University College of Medicine, Hershey, PA 17033 USA; 5 Department of Public Health Sciences, Pennsylvania State University College of Medicine, Hershey, PA 17033 USA; 6 Mass Spectrometry and Proteomics Facility, College of Medicine, Pennsylvania State University, Hershey, PA 17033 USA; 7 Department of Medicine, College of Medicine, Pennsylvania State University, Hershey, PA 17033 USA

**Keywords:** Breast cancer, Cancer prevention, Cancer, Chemical biology, Organic chemistry, Diseases, Chemistry

## Abstract

Docosahexaenoic acid (DHA) is known to inhibit breast cancer in the rat. Here we investigated whether DHA itself or select metabolites can account for its antitumor action. We focused on metabolites derived from the lipoxygenase (LOX) pathway since we previously showed that they were superior anti-proliferating agents compared to DHA; 4-OXO-DHA was the most potent. A lipidomics approach detected several LOX-metabolites in plasma and the mammary gland in rats fed DHA; we also identified for the first time, 4-OXO-DHA in rat plasma. In a reporter assay, 4-OXO-DHA and 4-HDHA were more effective activators of PPARɣ than DHA. In breast cancer cell lines, 4-OXO-DHA induced PPARɣ and 15-hydroxyprostaglandin dehydrogenase (15-PGDH) but inhibited the activity of NF-κB and suppressed PI3K and mTOR signaling. Because of the structural characteristics of 4-OXO-DHA (Michael acceptor), not shared by any of the other hydroxylated-DHA, we used MS and showed that it can covalently modify the cysteine residue of NF-κB. We have also shown that the chemopreventive effect of DHA is associated with significant reduction of PGE_2_ levels, in both rat mammary tumors induced by MNU and non-involved mammary tissues. Collectively, our results indicate that 4-OXO-DHA is the metabolite of choice in future chemoprevention studies.

## Introduction

While preclinical studies demonstrate the protective role of omega-3 fatty acids (n-3FA) against the development of breast cancer, numerous case–control, prospective cohort, biomarker and intervention studies have yielded mixed results on the tumor protective effect of n-3FA^1^. The inconsistent results are likely due to differences in the dose, duration, specific compounds used (DHA *vs.* eicosapentaenoic acid (EPA)), the ratio of n-3:n-6, and the target population tested (high *vs.* average risk), as well as the failure to address the complexity of fatty acid metabolism, and failure to differentiate between likely responders and non-responders in developing inclusion and exclusion criteria for participation^2^. Previous studies performed in vitro and in vivo model systems of mammary carcinogenesis demonstrated that DHA administered orally or mixed in the diet, is a superior chemopreventive agent to EPA^3–6^.

We have shown that only diets containing high ratios of n-3:n-6 (10:1 and 25:1) inhibited carcinogenesis, inhibited NF-κB ,and activated PPARɣ in mammary adenocarcinomas induced by *N*-methyl-*N*-nitrosourea (MNU) in the rat^7^. In fact, we showed that PPARɣ activation associated with downstream inhibition of NF-κB is central to the antitumor effect of a diet high in n-3FA in the rat^8^. Activation of PPARɣ can block NF-κB translocation to the nucleus and exert anti-inflammatory signaling^9–11^. Clearly our results and those reported in the literature support that PPARɣ and NF-κB pathways are, in fact, inter-dependent.

We also showed that plasma DHA levels peaked at a ratio of 5:1 of n-3:n-6FA which did not significantly reduce mammary tumors and mammary gland density, a parameter found to be predictive of cancer prevention^7^; in fact, levels of DHA were decreased in rats fed 10:1 and 25:1 ratios of n-3:n-6FA. These results suggest that DHA metabolites rather than DHA itself may account for the antitumor action of DHA. However, the role of DHA metabolism in the antitumor action of the parent compound remains largely undefined.

N-3 and n-6FA compete for the same metabolic enzymes such as cyclooxygenase (COX), cytochrome P450 and lipoxygenase (LOX) leading to metabolites with opposing effects on inflammation, cell proliferation and apoptosis (anti-tumorigenic with n-3FA; neutral or pro-tumorigenic with n-6)^1^. In contrast to metabolites derived from COX and cytochrome P450, studies on the anti-tumor action of LOX metabolites are scarce. We previously showed that DHA and two of its LOX-induced metabolites (4-OXO-DHA, 4-HDHA) significantly inhibited proliferation in multiple breast cancer cell lines^12^; however, the putative metabolite 4-OXO-DHA is far more potent than others. Furthermore, we showed that 4-OXO-DHA treatment preferentially affected triple-negative breast cancer cell lines^12^. However, it remained untested whether 4-OXO-DHA could be detected in vivo following DHA administration. Therefore, the studies reported herein were designed to answer the following questions: (1) Are DHA metabolites from LOX pathway (particularly 4-OXO-DHA) detectable in vivo in rats fed DHA? (2) Does the antitumor activity of the metabolites correlate with their ability to activate PPARɣ? (3) Can the superior antitumor action of 4-OXO-DHA in breast cancer cell lines be explained by a unique structure–activity relationship with regard to inhibition of NF-κB? (4) Do the differential effects of DHA on NF-κB and PPARɣ influence the levels of the proliferating agent PGE_2_ in mammary adenocarcinomas and non-involved tissues of rats treated with MNU?

## Results

### Lipidomic analysis of plasma and mammary tissues of rats fed DHA

**Figure 1:**
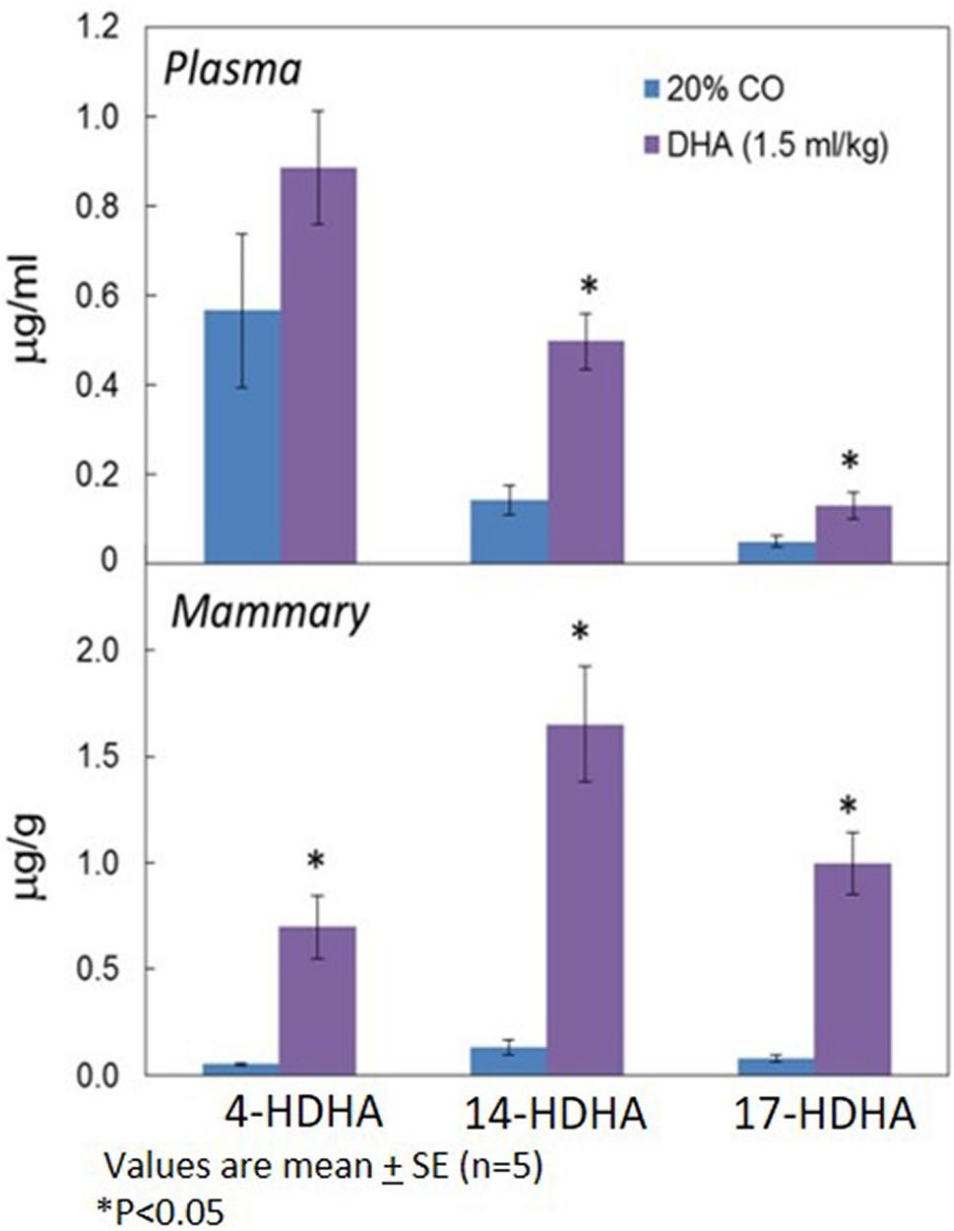
Levels of 4-HDHA, 14-HDHA, 17-HDHA in plasma and mammary tissues of rats treated with DHA using LC–MS/MS analysis.

**Figure 2:**
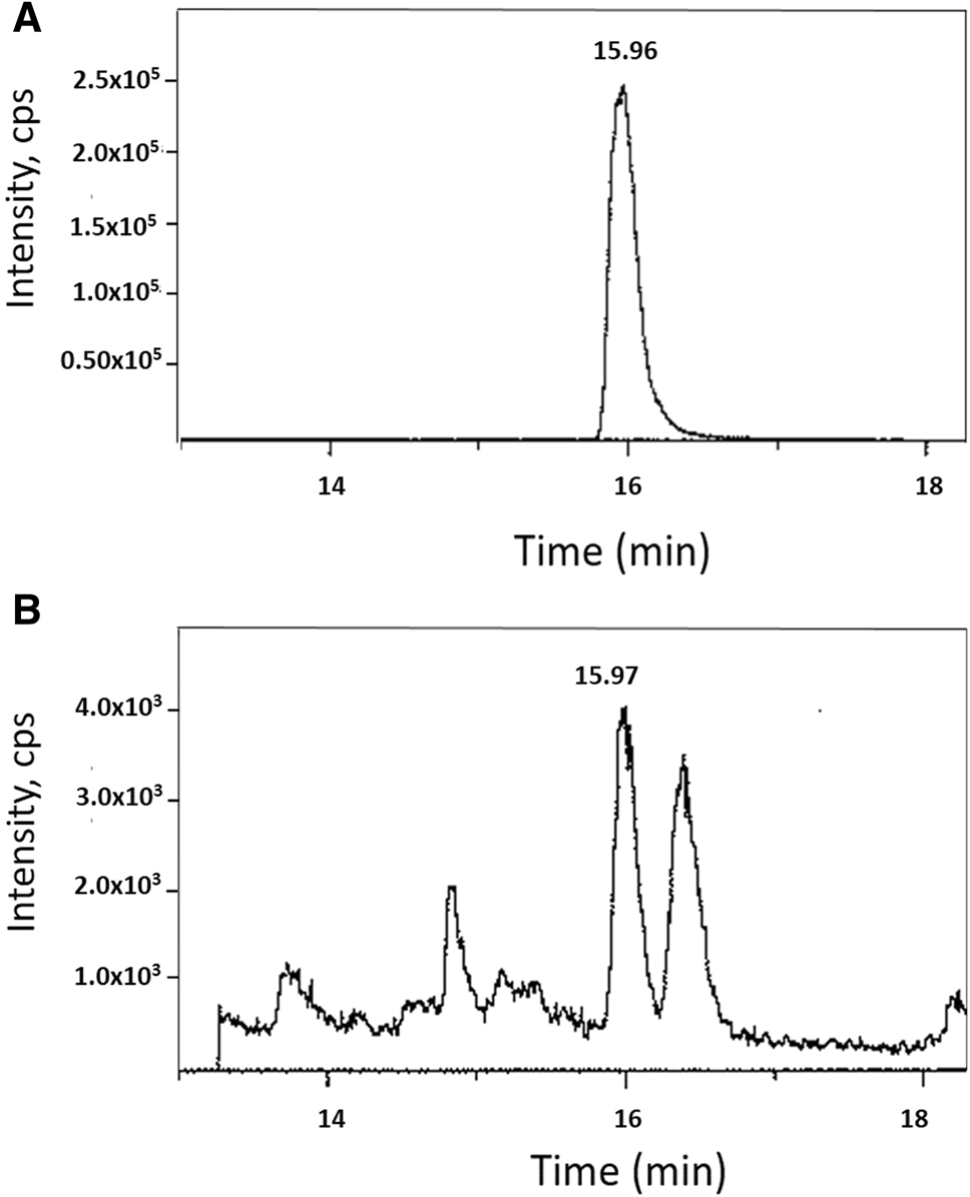
LC–MS/MS analysis of 4-OXO-DHA in rat plasma. ESI negative, transition m/e = 341 → 135. Panel **A** represents the synthetic standard (5 ng in ethanol), while Panel **B** represents the detection of 4-OXO-DHA in un-spiked plasma sample from rats that received DHA. In Panel **A**, 4-OXO-DHA (1 mg) was dissolved in 1 ml ethanol and farther diluted to yield 1 µg/1 ml solution; 5 µl was analyzed. In Panel **B**, plasma sample (500–700 µl) from each rat (n = 5) mixed with 500 µl of buffer (1 M sodium acetate, pH 6) and then was extracted 3 times with 2 ml of a mixture consisted with ethyl acetate:hexane:acetic acid (75:24:1). The extract was dried over sodium sulfate, filtered and then evaporated to dryness. The residue was reconstituted in 150 µl of ethanol and 5 µl was analyzed. CPS = counts per second.

In our previous study, following a direct comparison of DHA with two of its LOX-metabolites (4-HDHA, 4-OXO-DHA), we demonstrated that the metabolites are superior inhibitors of cell growth to that of the parent compound DHA using several breast cancer cells; 4-OXO-DHA was the most potent^12^. Although the synthesis of 4-OXO-DHA has been reported in the literature, to our knowledge, it has never been identified as a metabolite of DHA. Thus, in this study our initial goal was to identify LOX-metabolites (particularly 4-OXO-DHA) in rats administered DHA by oral gavage twice weekly until termination of the bioassay (*cf.* Materials and Methods) at a dose of 1.5 ml/kg b.w. We detected several other LOX-metabolites (4-, 14-, and 17-HDHA) in plasma and mammary fat pads of rats gavaged with DHA at 1.5 mL/Kg b.w. (Fig. 1). Levels of these metabolites (mean ± SE) in plasma (µg/mL) were 4-HDHA, 0.87 ± 0.26; 14-HDHA, 0.5 ± 0.06; 17-HDHA, 0.13 ± 0.03 and in the mammary tissues (µg/g) were 4-HDHA, 0.69 ± 0.15; 14-HDHA, 1.64 ± 0.27; 17-HDHA, 0.99 ± 0.15 of rats. In addition, we identified, for the first time, 4-OXO-DHA in plasma of rats fed DHA. LC–MS/MS analysis of the synthetic 4-OXO-DHA is shown in Fig. 2A, and 4-OXO-DHA metabolite in rat plasma is shown in Fig. 2B. To further confirm the identification of 4-OXO-DHA as a metabolite in plasma of rats fed DHA, co-chromatography of plasma samples spiked with the synthetic standard 4-OXO-DHA was conducted; the level of 4-OXO-DHA in plasma was 0.002 ± 0.001 µg/mL which is about 400 times lower than that of its precursor metabolite 4-HDHA. Limits of quantification, linear range and transitions used for each metabolite measured in this study are provided in Supplementary Table 1.

### The effects of DHA and LOX-metabolites on PPARɣ activity

**Figure 3:**
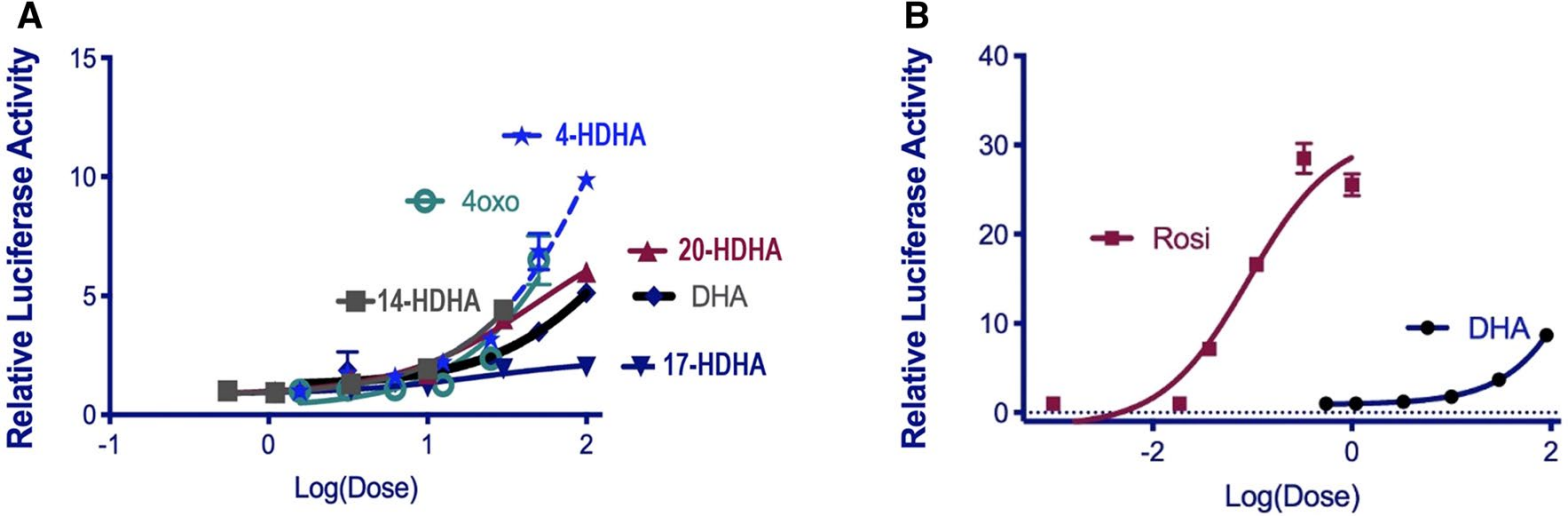
The effect of several ADM on PPARɣ activity. Human PPARɣ reporter cells were treated with various concentrations of ADM (0.5 to 100 μM final concentration in media, (**A**) or rosiglitazone (0.01 to 1 μM, [**B])** for 16 h. Data is derived from relative luciferase units (RLU) expressed relative to the vehicle control (DMSO, 0.1% v/v). Data represents mean ± SEM, n = 4, representative of at least two independent experiments.

Because of its superior anti-proliferative activity^12^, 4-OXO-DHA was selected to directly compare its effects with other metabolites at several doses (0.5–100 µM) on PPARɣ activation. We found that the PPARɣ agonist activity follows the order: 4-OXO-DHA ≃ 4-HDHA ≃ 14-HDHA > DHA > 20-HDHA > 17-HDHA (Fig. 3A,B).

### The Effects of 4-OXO-DHA on NF-κB-DNA Binding

**Figure 4:**
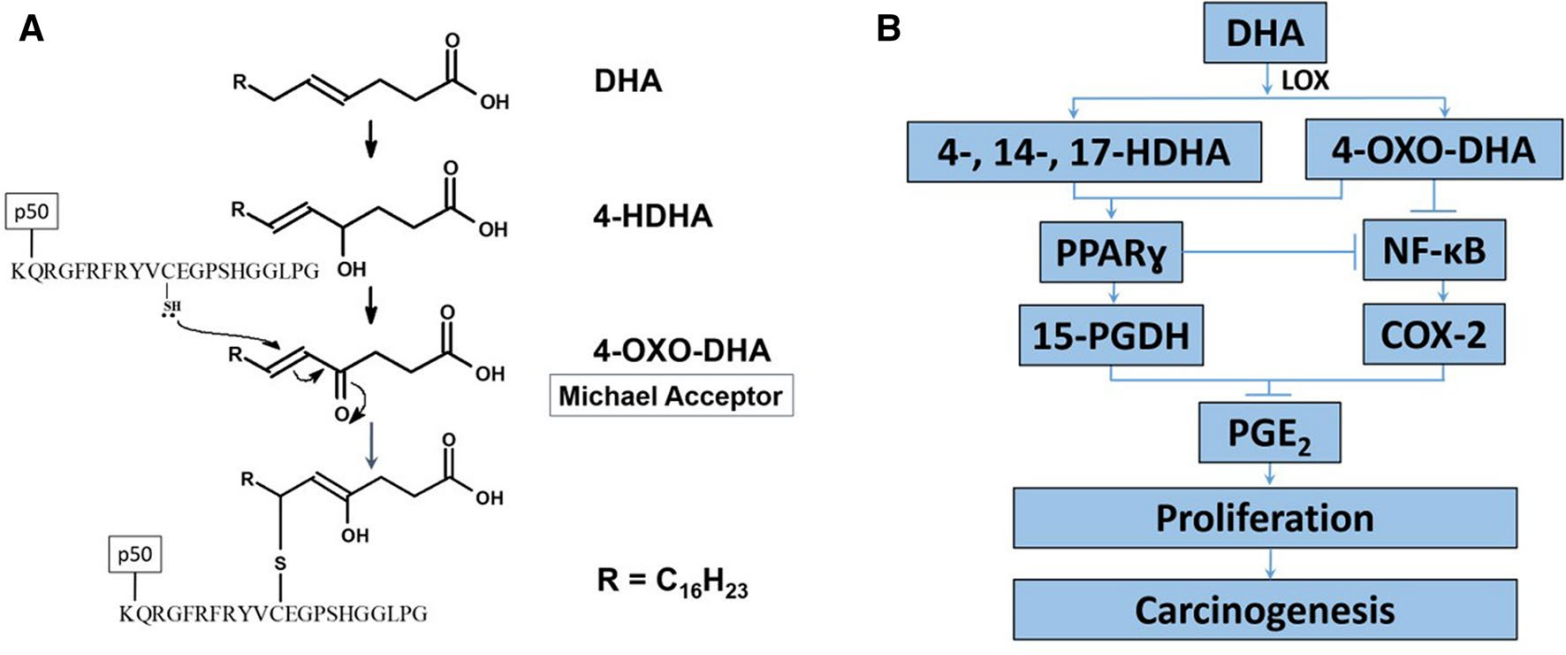
**(A)** NF-κB as a target for antitumor action of mammary carcinogenesis by DHA and metabolites (4-OXO-DHA). **(B)** Presentation of our working hypothesis.

**Figure 5:**
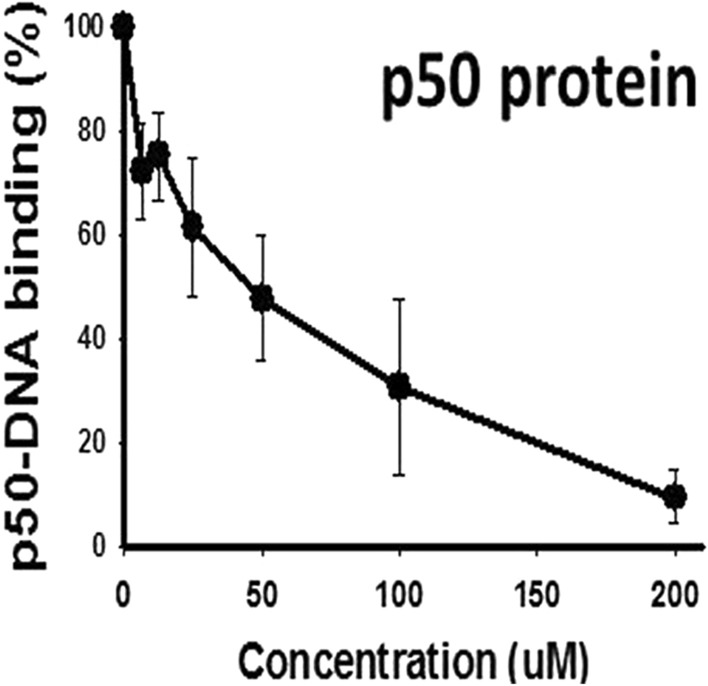
Inhibition of recombinant NF-κB (p50) DNA binding by 4-OXO-DHA.

**Figure 6:**
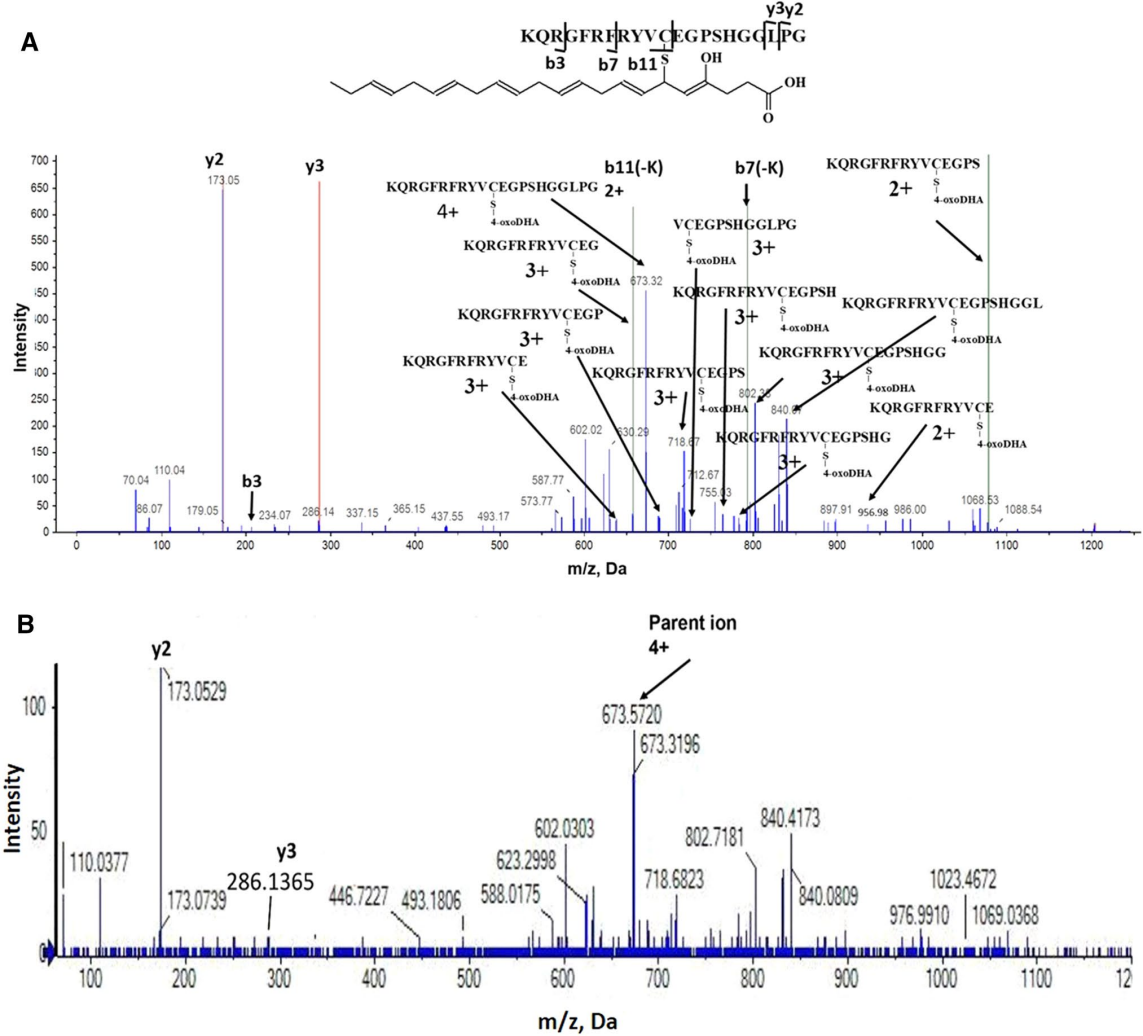
MS/MS spectrum of (**A**) the peptide (2,689.8 Da) containing the 4-OXO-DHA-modified sequence of the active site (KQRGFRFRYVCEGPSHGGLPG) of p50 and (**B**) the fragmentation pattern following the incubation of 4-HDHA with the same peptide.

To test our chemical hypothesis (Fig. 4A) and provide structural basis on the role of 4-OXO-DHA as an inhibitor of NF-κB-DNA binding, in Fig. 5, we showed that at various concentrations (5, 10, 25, 50, 100, 200 µM), 4-OXO-DHA dose-dependently inhibited NF-κB-DNA binding. Furthermore, we demonstrated that 4-OXO-DHA modified a peptide containing the sequence in the active site of p50 via the formation of a covalent bond to the cysteine residue by MS/MS analysis (Table 1 and Fig. 6A). Peaks corresponding to the monoisotopic M° mass of the peptide itself (2347.175) were seen in control and treated samples, while a peak corresponding to the M° mass of the peptide plus the mass of the 4-OXO-DHA (341.21) of 2688.385 was only seen in the 4-OXO-DHA treated peptide sample. We had also detected the fragment containing unmodified sequence of QRGFRFRYVC (labelled as b11(-K) in Fig. 6A); in addition, all other identified fragments containing cysteine residue were all modified with 4-OXO-DHA. Although the 4-OXO-DHA modified QRGFRFRYVC or KQRGFRFRYVC were not observed, the 4-OXO-DHA modified KQRGFRFRYVCE was present. Because it is unlikely that Glu would be modified by 4-OXO-DHA, our results support that 4-OXO-DHA (an electrophilic Michael acceptor) can covalently bind to the peptide containing the sequence of the active site of NF-κB p50 protein to form a covalent linkage with the nucleophilic Cysteine-S^62^ residue (marked in bold and italic in Table 1). To further support our hypothesis that the inhibition of NF-κB-DHA binding by 4-OXO-DHA is due to its structural characteristic as a Michael acceptor, which is not shared by other mediators, we incubated 4-HDHA with the above mentioned peptide containing the sequence of the active site of the NF-κB p50 protein under identical reaction conditions to those of 4-OXO-DHA. The results showed that the fragmentation pattern of the peak with m/z of 2689 (Fig. 6B) was different from that observed in the case of 4-OXO-DHA modified peptide (Fig. 6A); clearly fragments containing 4-OXO-DHA modified cysteine residue were not observed with 4-HDHA supporting our hypothesis that such covalent binding of the cysteine residue is specific to 4-OXO-DHA and not to other mediators such as 4-HDHA.

**Table 1:** Identification of 4-OXO-DHA modified peptide containing the active site sequence (KQRGFRFRYV***C***EGPSHGGLPG) of p50 by MS/MS (cysteine is labeled in bold and Italic).

Fragments with modification	Fragments without modification
KQRGFRFRYV***C***E	KQR
KQRGFRFRYV***C***EG	QRGFRF
KQRGFRFRYV***C***EGP	QRGFRFRYVC
KQRGFRFRYV***C***EGPS	LPG
KQRGFRFRYV***C***EGPSH	PG
KQRGFRFRYV***C***EGPSHG	
KQRGFRFRYV***C***EGPSHGG	
KQRGFRFRYV***C***EGPSHGGL	
KQRGFRFRYV***C***EGPSHGGLPG	
V***C***EGPSHGGLPG

### The Effects of 4-OXO-DHA on Molecular Targets that can account for Cell Growth Inhibition

Since 4-OXO-DHA is now unequivocally identified in plasma of rats fed DHA and it is the most potent metabolite to inhibit cell growth^12^ and has a unique structural characteristics of inhibition of NF-κB, further studies were performed to more widely explore the magnitude of its effects on different molecular targets using three different molecular subtypes of human breast csancer cell lines (Table 2). PPARɣ and 15-PGDH were induced, the activity of NF-κB (p65^ser536^/NF-κB) was suppressed, mTOR signaling was inhibited (pP70S6K^Thr389/P7056^), and PI3K signaling was reduced (pAKt^ser473/^AKt).

**Table 2:** The effect of 4-OXO-DHA on molecular targets in breast cancer cell lines.

	BT-474		SK-BR-3		MDA-MB-468	
ER	+		–		–	
PR	+		–		–	
HER2	+		+		–	
4-OXO-DHA (uM)	0	25	0	25	0	25
^a^PPARy (AUC)	45 ± 1	57 ± 1*	94 ± 2	124 ± 3*	126 ± 1	153 ± 2*
^a^15-PGDH (AUC)	50 ± 2	68 ± 5*	70 ± 2	86 ± 5*	58 ± 1	72 ± 2*
^b^pNF-κB (AUC) p65^Ser536^	0.65 ± 0.04	0.45 ± 0.03*	0.45 ± 0.02	0.50 ± 0.02	2.38 ± 0.12	1.28 ± 0.08*
^b^pAkt^Ser473^ (AUC)	3.29 ± 0.13	0.78 ± 0.03*	3.23 ± 0.23	1.01 ± 0.08*	7.68 ± 0.29	6.50 ± 0.26*
^b^pP70S6K^Thr389^ (AUC)	0.26 ± 0.01	0.10 ± 0.01*	0.55 ± 0.17	0.10 ± 0.01*	0.93 ± 0.03	0.67 ± 0.03*

^a^Normalized to GAPDH.

^b^Normalized to total amount of that protein detected by Western Blotting.

^c^arbitarary units of chemiluminesence, AUC.

Vaules with *, significantly different, *p* < 0.05.

### DHA reduces tissue level of PGE_2_ in normal breast and breast cancer tissue

In this experiment, our goal was to test our hypothesis (Fig. 4B) that the differential effect of DHA on NF-κB and PPARɣ will impact the levels of PGE_2_. We showed that in subsets of mammary carcinomas and non-involved mammary tissues (n = 6 rats/group), PGE_2_ levels were significantly lower in DHA treated rats; PGE_2_ levels were 2.3 times lower (18 vs. 41 nM) in mammary carcinoma, and 5 times lower in non-involved mammary tissue (2.8 vs. 15 nM) using factorial analysis of variance (p < 0.05). Consistent with previous studies^3,4^, DHA administration inhibited tumor growth as shown by a reduction in tumor multiplicity (mean ± SE: DHA, 1.69 ± 0.26; corn oil (control), 2.55 ± 0.32). Throughout the duration of the bioassay, we found no significant difference in body weight (Supplementary Figure S1) between the two groups.

## Discussion

In our previous study^12^, using multiple breast cancer cell lines with different molecular subtypes, we clearly showed that 4-OXO-DHA was a superior anti-proliferating metabolite to that of DHA and 4-HDHA. Therefore, the focus of the present study is to determine whether 4-OXO-DHA can be identified as a metabolite of DHA in vivo, to examine its effects on critical molecular targets of mammary carcinogenesis in breast cancer cell lines in vitro and to elucidate the structural basis that can account for its inhibition of NF-κB. We showed that levels of LOX-metabolites were significantly higher in the mammary gland and plasma of rats administered DHA orally than in untreated rats although the increase in the plasma level of 4-HDHA derived from 5-LOX activation was not significant. It has been reported that 5-LOX requires accessory proteins for maximal activity such as 5-LOX Activation Protein (FLAP) embedded in the nuclear membrane and a cytosolic-coactosin-like protein^13,14^; protein/protein interaction (5-LOX/FLAP) in vivo is likely to be the driving force for the formation of 4-HDHA. These results demonstrate that these LOX-metabolites can reach the target organ (mammary gland) of rats administered DHA. DHA can be enzymatically converted by platelets, basophils, and liver microsomes to several hydroxylated derivatives^15–17^. Using LC–MS/MS-based lipidomics, Saphieha et al. identified 4-HDHA as a major metabolite in serum and retina of mice fed n-3FA diet^18^. Weiss et al. detected 17-HDHA in human milk during the first ten days of lactation^19^. Although other hydroxylated metabolites derived from DHA have been identified in various biological fluids^20^, to our knowledge, 4-OXO-DHA has never been detected in animals fed n-3FA or in humans. Under our experimental conditions, we unequivocally identified for the first time 4-OXO-DHA in plasma of rats fed DHA. Given 4-OXO-DHA is the metabolite with the strongest antitumor activity and preferentially affected triple-negative breast cancer cell lines^12^, its identification is an important finding of this report.

In a previous study, Itoh et al. demonstrated that the PPARɣ receptor can (1) simultaneously bind two fatty acids (non-covalently) and (2) couple covalently with 4-OXO-DHA^21^. To discover novel PPARɣ agonists, additional studies showed that 4-HDHA, 4-OXO-DHA and 17-OXO-DHA are strong PPARɣ transcriptional activators^21–23^. Our results showed that the activities of 4-OXO-, 4-HDHA, and 14-HDHA while superior to that of DHA were comparable among themselves on PPARɣ activation; however, these results do not explain the superior antitumor action of 4-OXO-DHA^12^. Moreover, 17-OXO-DHA hasn’t been identified as a metabolite of DHA. Therefore, to explain the superior antitumor action of 4-OXO-DHA, we hypothesized in Fig. 4A the structural basis for the inhibitory effect of 4-OXO-DHA on NF-κB; supporting our hypothesis we showed that 4-OXO-DHA can covalently bind to cysteine moiety in NF-κB and such binding may account for the inhibition of this transcriptional factor. To further support our hypothesis, we examined the effect of 4-HDHA which lacks a Michael acceptor moiety on NF-κB-DNA binding; the MS results showed that the binding of the cysteine residue was evident with 4-OXO-DHA but not with 4-HDHA.

Our in vitro studies using three breast cancer cell lines with different molecular subtypes were aimed at determining the effect of 4-OXO-DHA on additional molecular markers besides PPARɣ and NF-κB; the results are consistent with our previous in vivo studies demonstrating that a diet rich in n-3FA altered similar molecular targets in a manner consistent with breast cancer prevention in the rat^7,8^. Convincing evidence indicates that n-3FA exert biological effects, in part, via the activation of PPAR receptors which regulate transcription of genes involved in cell proliferation, cell survival, apoptosis and cellular metabolism^24^. The mechanisms that can account for these effects include PPARɣ-dependent modulation of proteins involved in the above-mentioned cellular processes^25^. A decrease in phosphorylation of AKT, as regulated by PPARɣ^26^ is associated with reduced mTOR activity^27^. Furthermore, decreased levels of phosphorylated pP70S6K, a substrate of mTOR, can regulate cell proliferation, cell survival and other aspects of cellular metabolism^28^.

We further hypothesized that the signaling-effects reported here should lead to a reduction in PGE_2_ as proposed in Fig. 4B. We indeed showed that DHA reduced the levels of PGE_2_ in mammary tumors as well as in non-involved mammary tissues; these results are consistent with the activation of PPARɣ and inhibition of NF-κB resulting in suppression of COX-2^29,30^. Reduction of tissue levels of PGE_2_ is also expected from DHA stimulation of 15-PGDH, a major catabolic pathway of PGE_2_.

In our in vitro studies, DHA was used at levels comparable to those published in the literature by us and others^5,6,12^. Thus, for comparison we utilized LOX-metabolites at doses equal to that of DHA. However, we fully recognize that the concentrations of LOX-metabolites utilized in this study were much higher than those detected in plasma and mammary tissues of rats orally administered DHA. Nevertheless, these LOX-metabolites were measured in vivo at a single time point which is likely at their elimination phase and thus it is considered a limitation of our study. Clearly, future studies should focus on determining pharmokinetic parameters (Cmax, Tmax, t_1/2_) of the various LOX-metabolites; such information will be essential prior to the design of future preclinical chemoprevention studies.

In summary, our results provide mechanistic insights on the effects of DHA and its LOX-metabolites on several critical molecular targets in the development of mammary carcinogenesis. Furthermore, our results suggest that cysteine moiety (a nucleophile) within NF-κB-p50 can covalently bound to 4-OXO-DHA (an electrophilic Michael acceptor). However, it is essential to fully understand the metabolism of DHA and how the various metabolites, individually and in combination alter cellular and molecular targets critical in the prevention of breast cancer. Furthermore, it is also important to consider metabolites not only derived from LOX pathway but also those derived from CYP450 and COX pathways. Moreover, a comparison of the chemopreventive efficacy of dietary DHA with 4-OXO-DHA in well-defined animal models of mammary carcinogenesis will be pursued; such a comparison will provide ample opportunity to examine the effects of these agents on molecular targets including PGE_2_ and an established marker of cell proliferation (e.g. Ki67) in the rat mammary tumor as well as non-involved tissue.

## Materials and methods

### The effects of DHA on mammary carcinogenesis, levels of PGE_2_ and identification of its metabolites in plasma, mammary tumors and mammary tissue in the rat

Animal studies were approved by the Institutional Animal Care and Use Committee of the Pennsylvania State University College of Medicine and experiments were performed in accordance with the relevant guidelines and regulations. MNU was obtained from MRI Global Chemical Carcinogen Repository, Kansas City, MO. Modified AIN-76A diet (20% corn oil) was prepared in our laboratory biweekly^31^ from ingredients obtained from Research Dyets, and stored at 4° until use; diet was provided *ad. libitum* every 2–3 days. Animal rooms were maintained at 22 ± 1 °C with 50% relative humidity and a 12-h light/12-h dark cycle. At 21 days of age female Sprague–Dawley rats (Charles River Laboratories International) were injected with 50 mg MNU/kg body weight, i.p. as previously reported^7^. Seven days following MNU injection, one group of rats (n = 25) was orally administered with DHA (1.5 mg/kg body weight, Bizen Chemical Co., LTD, Okayama Japan) twice weekly until termination of the bioassay (8 weeks after MNU administration); the dose of DHA is comparable to that reported in the literature^3^. A second group of rats (n = 25) was treated with MNU and fed the same diet but without DHA administration. All rats were weighed weekly and palpable tumors were recorded and measured weekly until sacrifice. Following an overnight fast, rats were euthanized via inhalation of carbon dioxide. Blood was obtained from the retro-orbital sinus, and plasma was prepared by centrifugation. Following cervical dislocation, mammary glands were collected, measured, fixed in 10% neutral buffered formalin, processed and paraffin embedded for histological analysis according to previously reported criteria^32^. Cancer multiplicity (number of adenocarcinomas/rat following square root transformation of the count data), body weights, and PGE_2_ levels (determined by ELISA, Cayman Chemical, Ann Arbor, MI) were statistically evaluated by ANOVA^7^.

### Lipidomic analysis of DHA metabolites derived from LOX pathway

Rat plasma between 500 and 700 µl (n = 5/group) was mixed with 500 µl of buffer (1 M pH 6 sodium acetate + 5% methanol). Each sample was then extracted three times with 2 ml of a solution containing ethyl acetate:hexane:acetic acid = 75:24:1. The extract was dried, filtered and evaporated to dryness. The residue was reconstituted in 150 µl ethanol, and then filtered using Ultrafree—MC-HV filters (Millipore) at 6800 rcf for 5 min prior to analysis. Mammary tissues (n = 5/group) of rats were homogenized and processed as described above.

Metabolites were analyzed by negative ion LC–MS/MS electrospray (ABSciex Q Trap 4500) using an Agilent Zorbax 2.1 × 150 mm C18 column. A gradient program was run from 90% solvent A (water) to 90% solvent B (acetonitrile) in 10 min and the final condition was held for 10 min. The flow rate was 0.25 ml/min. The ion spray voltage was -4500, the temperature was 450 °C, and the declustering potential was -55 V. Deuterated DHA was used as an internal standard for quantification of LOX-metabolites. 4-HDHA eluted at 15.7 min, 14-HDHA at 14.23 min, and 17-HDHA at 18.55 min. The lipidomic analysis of 4-OXO-DHA in plasma of rats fed DHA was determined but the MS conditions were modified (DP = -85 and temp = 300); 4-OXO-DHA was eluted at 15.97 min. Detection limit and transitions used in this study are described in Supplementary Table 1.

### The effect of DHA and LOX-metabolites on PPARɣ activity

Human PPARɣ reporter assay system was purchased from INDIGO Biosciences, Inc. (State College, PA). Assays were performed according to the manufacturer’s instructions using Rosiglitazone as a reference agonist. DHA and DHA metabolites (4-OXO-DHA, 4-HDHA, 14-HDHA, 20-HDHA and 17-HDHA) were either obtained commercially and/or synthesized by previously described methods^21,22,33^. The purities of these metabolites based on HPLC analysis exceeded 98%. Non-linear regression to determine EC_10_ and peak activity was performed in GraphPad Prism (San Diego, CA).

### The effects of 4-OXO-DHA on molecular targets that can account for cell growth inhibition

The goal of the present study was to elucidate the molecular targets that can account for growth inhibition by 4-OXO-DHA (25 µM, 72 h exposure) using breast cancer cells with different molecular subtypes [ER, PR, Her-2: + , + , + (BT-474); −, −, + (SK-BR-3); −, −, −(MDA-MB-464)]. Breast cancer cell lines were purchased from ATCC, Manassas, Virginia. Antibodies were obtained commercially and nanoimmunocapillary electrophoresis was performed as described previously^34^.

### Structural basis for the inhibition of NF-κB-DNA binding by 4-OXO-DHA

Employing our experimental approach^35^, pure p50 protein (Active Motif North America, Carlsbad, CA) at a concentration of 3 nM was incubated with various concentrations of 4-OXO-DHA (6.25, 12.5, 25, 50, 100, 200 µM) at ambient temperature for 30 min with 10% DMSO as vehicle without the addition of 4-OXO-DHA in control samples. This experiment was conducted in triplicate and the incubation mixture was then added into a 96-well plate that had been coated with immobilized oligonucleotide containing a consensus-binding site for NF-κB^35^. Following the outcome of this study, we hypothesize (Scheme 1A) that 4-OXO-DHA (a Michael acceptor) can act as an electrophile and bind covalently to cysteine moiety (as a nucleophile) within NF-κB. Thus, we incubated 0.213 µmol/mL of the NF-κB (p50) active site peptide (KQRGFRFRYVCEGPSHGGLPG, from Biomatik, Ontario, Canada) with or without 4-OXO-DHA or 4-HDHA (5 µmol/mL) for 1 h at room temperature in a volume of 200 ul. One ul of each Treated or Control sample was injected onto a 350 µm × 0.5 mm ChromXP C18-CL 3 µm 120 Å trap column, then eluted onto a 75 µm × 15 cm ChromXP 3C18-CL 3 µm 120 Å nanoflow column at a flow rate of 300 nl/minute using an Eksigent NanoLC-Ultra-2D Plus system. The column eluate was nanosprayed into a Sciex 5600 + Triple-TOF mass spectrometer with gas settings of Ion source gas1 set at 3, gas2 set at zero, and Curtain Gas set at 25. The Ion Spray Voltage Floating (ISVF) was set at 2550 V, and the Interface Heater Temperature (IHT) was set at 150 °C. For the gradient elution, Buffer A was 0.1% formic acid in water, and Buffer B was acetonitrile and 0.1% formic acid, and both buffers were nitrogen sparged before use to degas. The peptide peak(s) were eluted with a gradient starting at 95% Buffer A, and 5% Buffer B, and changing linearly to 94.6% A and 5.4% B at 1 min, then linearly to 68% A and 32% B at 16 min, then linearly to 20% A and 80% B at 18 min, where it was held isocratically until 24 min. From 24 to 25 min the gradient was linearly switched back to starting conditions, 95% A and 5% B, and held isocratically until the next injection at 30 min. Duty cycle was 2.75 s, with parent scans of each eluate time slice taken for 250 mseconds, then 2.5 s of MS/MS scans were taken of all peaks appearing above a minimum threshold. Up to 50 MS/MS scans of 50 ms each were taken over that 2.5 s period, with proportionally more time allocated per MS/MS scan if fewer than 50 peaks appeared in the parent scan.

## Supplementary Information

Supplementary Information.

## Data Availability

The datasets generated during and/or analyzed during the current study are available from the corresponding author on reasonable request.

## References

[1] Signori C (2011). Chemoprevention of breast cancer by fish oil in preclinical models: trials and tribulations. Cancer Res..

[2] El-Bayoumy K, Manni A (2020). Customized prevention trials could resolve the controversy of the effects of omega-3 fatty acids on cancer. Nutr. Cancer.

[3] Noguchi M (1997). Chemoprevention of DMBA-induced mammary carcinogenesis in rats by low-dose EPA and DHA. Br. J. Cancer.

[4] Yuri T (2003). Dietary docosahexaenoic acid suppresses N-methyl-N-nitrosourea-induced mammary carcinogenesis in rats more effectively than eicosapentaenoic acid. Nutr. Cancer.

[5] Kang KS (2010). Docosahexaenoic acid induces apoptosis in MCF-7 cells in vitro and in vivo via reactive oxygen species formation and caspase 8 activation. PLoS ONE.

[6] Rahman MM, Veigas JM, Williams PJ, Fernandes G (2013). DHA is a more potent inhibitor of breast cancer metastasis to bone and related osteolysis than EPA. Breast Cancer Res. Treat..

[7] Zhu Z (2011). Mammary gland density predicts the cancer inhibitory activity of the N-3 to N-6 ratio of dietary fat. Cancer Prev. Res. (Phila).

[8] Jiang W (2012). Identification of a molecular signature underlying inhibition of mammary carcinoma growth by dietary N-3 fatty acids. Cancer Res..

[9] Turk HF, Chapkin RS (2013). Membrane lipid raft organization is uniquely modified by n-3 polyunsaturated fatty acids. Prostaglandins Leukot Essent Fatty Acids.

[10] Calder PC (2013). n-3 fatty acids, inflammation and immunity: new mechanisms to explain old actions. Proc Nutr. Soc..

[11] Fabian CJ, Kimler BF, Hursting SD (2015). Omega-3 fatty acids for breast cancer prevention and survivorship. Breast Cancer Res..

[12] Pogash TJ (2015). Oxidized derivative of docosahexaenoic acid preferentially inhibit cell proliferation in triple negative over luminal breast cancer cells. Vitro Cell Dev. Biol. Anim..

[13] Mitra S, Bartlett SG, Newcomer ME (2015). Identification of the substrate access portal of 5-lipoxygenase. Biochemistry.

[14] Czapski GA, Czubowicz K, Strosznajder JB, Strosznajder RP (2016). The lipoxygenases: their regulation and implication in Alzheimer's disease. Neurochem. Res..

[15] VanRollins M, Murphy RC (1984). Autooxidation of docosahexaenoic acid: analysis of ten isomers of hydroxydocosahexaenoate. J. Lipid Res..

[16] Aveldano MI, Sprecher H (1983). Synthesis of hydroxy fatty acids from 4, 7, 10, 13, 16, 19-[1-14C] docosahexaenoic acid by human platelets. J. Biol. Chem..

[17] Corey EJ, Shih C, Cashman JR (1983). Docosahexaenoic acid is a strong inhibitor of prostaglandin but not leukotriene biosynthesis. Proc. Natl. Acad. Sci. USA.

[18] Sapieha P (2011). 5-Lipoxygenase metabolite 4-HDHA is a mediator of the antiangiogenic effect of omega-3 polyunsaturated fatty acids. Sci. Transl. Med..

[19] Weiss GA (2013). High levels of anti-inflammatory and pro-resolving lipid mediators lipoxins and resolvins and declining docosahexaenoic acid levels in human milk during the first month of lactation. Lipids Health Dis..

[20] Kuda O (2017). Bioactive metabolites of docosahexaenoic acid. Biochimie.

[21] Itoh T (2008). Structural basis for the activation of PPARgamma by oxidized fatty acids. Nat. Struct. Mol. Biol..

[22] Itoh T, Murota I, Yoshikai K, Yamada S, Yamamoto K (2006). Synthesis of docosahexaenoic acid derivatives designed as novel PPARgamma agonists and antidiabetic agents. Bioorg. Med. Chem..

[23] Egawa D, Itoh T, Akiyama Y, Saito T, Yamamoto K (2016). 17-OxoDHA is a PPARalpha/gamma dual covalent modifier and agonist. ACS Chem. Biol..

[24] Peters JM, Shah YM, Gonzalez FJ (2012). The role of peroxisome proliferator-activated receptors in carcinogenesis and chemoprevention. Nat. Rev. Cancer.

[25] Frohlich E, Wahl R (2015). Chemotherapy and chemoprevention by thiazolidinediones. Biomed. Res. Int..

[26] Nickkho-Amiry M, McVey R, Holland C (2012). Peroxisome proliferator-activated receptors modulate proliferation and angiogenesis in human endometrial carcinoma. Mol. Cancer Res..

[27] Gwinn DM (2008). AMPK phosphorylation of raptor mediates a metabolic checkpoint. Mol. Cell.

[28] Ghayad SE, Cohen PA (2010). Inhibitors of the PI3K/Akt/mTOR pathway: new hope for breast cancer patients. Recent Pat. Anticancer Drug Discov..

[29] Rose DP, Connolly JM (1997). Dietary fat and breast cancer metastasis by human tumor xenografts. Breast Cancer Res. Treat..

[30] D'Eliseo D, Velotti F (2016). Omega-3 fatty acids and cancer cell cytotoxicity: implications for multi-targeted cancer therapy. J. Clin. Med..

[31] Manni A (2014). Influence of omega-3 fatty acids on Tamoxifen-induced suppression of rat mammary carcinogenesis. Int. J. Cancer.

[32] Goodman, D. G, Maronpot, R. R., Newberne, P. M., Popp, J. A. Squire, R. A. Proliferative and selected other lesions in the liver of rats. G1-5. In *Guides for Toxicologic Pathology* (STP/ARP/AFIP, Washington DC, 1994).

[33] Itoh T, Saito T, Yamamoto Y, Ishida H, Yamamoto K (2016). Gram scale synthesis of specialized pro-resolving mediator 17(S)-HDHA using lipoxygenase enhanced by water-soluble reducing agent TCEP. Bioorg. Med. Chem. Lett..

[34] Thompson HJ, McGinley JN, Neil ES, Brick MA (2017). Beneficial effects of common bean on adiposity and lipid metabolism. Nutrients.

[35] Chen KM (2007). Inhibition of nuclear factor-kappaB DNA binding by organoselenocyanates through covalent modification of the p50 subunit. Cancer Res..

