# Supplementary material for: Lipoxygenase catalyzed metabolites derived from docosahexaenoic acid are promising antitumor agents against breast cancer
Source: Sci Rep. 2021 Jan 11;11:410. doi: 10.1038/s41598-020-79716-x (PMC7801725; doi:10.1038/s41598-020-79716-x)
Supplement: Supplementary file 1 — Supplementary Information. [file 41598_2020_79716_MOESM1_ESM.docx]

**Supplementary File Information for:**

Lipoxygenase Catalyzed Metabolites Derived from Docosahexaenoic Acid are Promising Antitumor Agents against Breast Cancer

Kun-Ming Chen^1^, Henry Thompson^2^, John P. Vanden-Heuvel^3^, Yuan-Wan Sun^1^, Neil Trushin^5^, Cesar Aliaga^1^, Krishne Gowda^4^, Shantu Amin^4^, Bruce Stanley^6^, Andrea Manni^7^ and Karam El-Bayoumy^1,^*

^1^ Department of Biochemistry and Molecular Biology, Pennsylvania State University, College of Medicine, Hershey, Pennsylvania 17033, USA

^2^ Cancer Prevention Laboratory, Colorado State University, Fort Collins, Colorado 85023, USA

^3^ Department of Veterinary and Biomedical Sciences, Pennsylvania State University, University Park, Pennsylvania 16802, USA

^4^ Department of Pharmacology, Pennsylvania State University College of Medicine, Hershey, Pennsylvania 17033, USA

^5^ Department of Public Health Sciences, Pennsylvania State University College of Medicine, Hershey, Pennsylvania 17033, USA

^6^ Mass Spectrometry and Proteomics Facility, Pennsylvania State University, College of Medicine, Hershey, Pennsylvania 17033, USA

^7^ Department of Medicine, Pennsylvania State University, College of Medicine, Hershey, Pennsylvania 17033, USA

***** Correspondence: [kee2@psu.edu](mailto:kee2@psu.edu); Tel: 717-531-1079, Fax: 717-531-0002

**Supplementary Legends**

**Supplementary Figure 1.** Body weight of female Sprague-Dawley rats throughout the duration of the bioassay. Rats were injected with MNU (50 mg/kg body weight) at 21 days of age and then fed a modified AIN 76A diet that contains 20% corn oil. Seven days after carcinogen treatment, one group of rats was given DHA by oral gavage (1.5 ml/kg body weight) and the other group was not treated (note: DHA is a liquid and thus was needed for a vehicle).


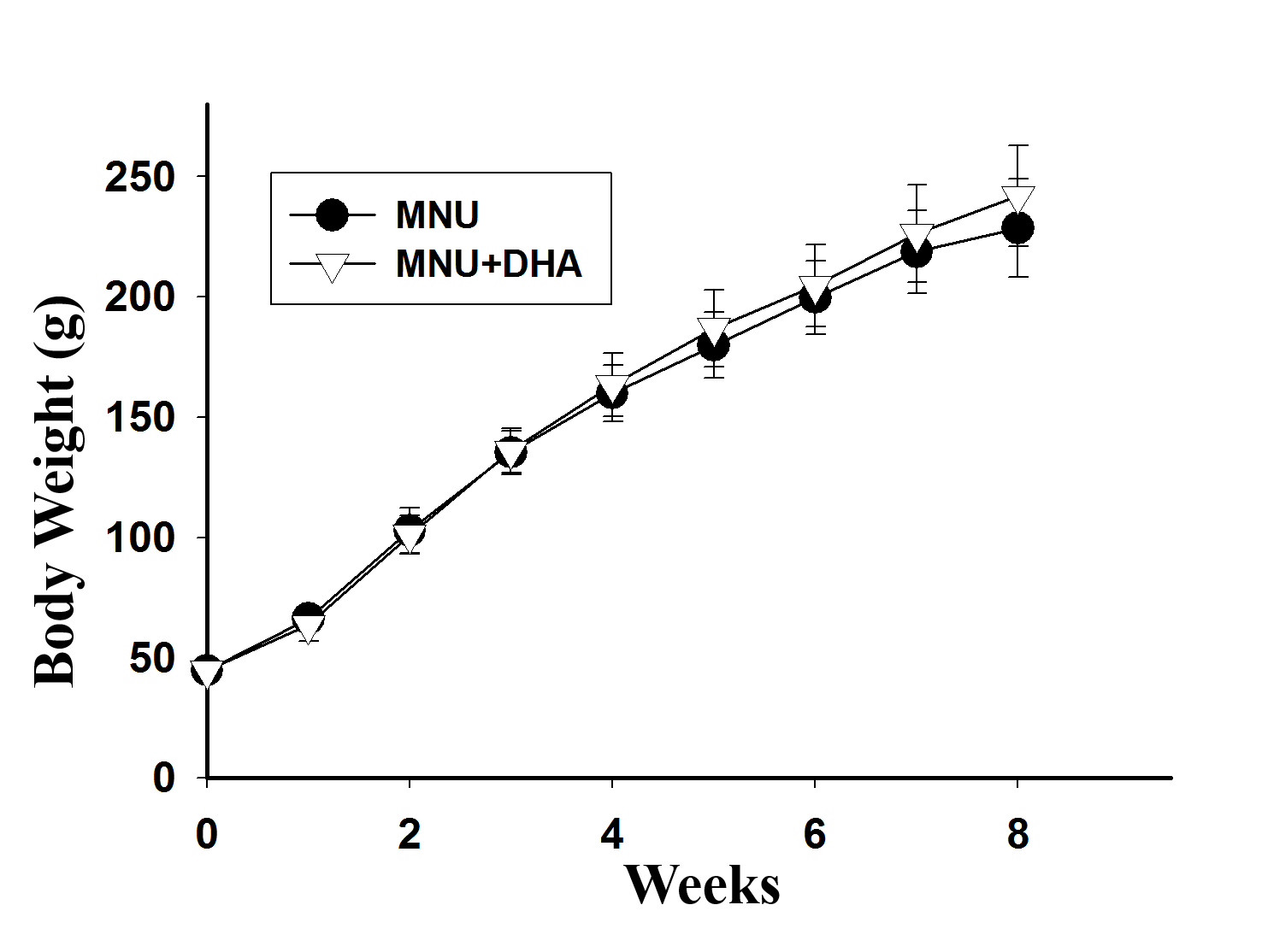


**Supplementary Table 1. Transitions, detection limits, regression coefficients and ranges of standard curves for the detection of DHA metabolites by HPLC-MS/MS.**

| Metabolite | Transition | Detection  Limit | R^2^ | Standard Curve Range (pg) |
| --- | --- | --- | --- | --- |
| 4-HDHA | **343.2 to 101-2** | **2.5** | **0.989** | **2-40** |
| 14-HDHA | **343.2 to 161.2** | **0.5** | **0.994** | **20-400** |
| 17-HDHA | **343.2 to 201.2** | **5.0** | **0.992** | **4-80** |
| 4-OXO-DHA | **341.2 to 135.1** | **10.1*** | **ND*** | **ND*** |

*Detection limit is an estimate because the injection of 4-OXO-DHA was done once.

ND = Not done
